# Application of FTIR spectroscopy for traumatic axonal injury: a possible tool for estimating injury interval

**DOI:** 10.1042/BSR20170720

**Published:** 2017-07-21

**Authors:** Ji Zhang, Ping Huang, Zhenyuan Wang, Hongmei Dong

**Affiliations:** 1 Shanghai Key Laboratory of Forensic Medicine, Institute of Forensic Science, Ministry of Justice, Shanghai 200063, China; 2 Department of Forensic Pathology, College of Forensic Medicine, Xi’an Jiaotong University, Xi’an 710061, Shaanxi, China; 3 Department of Forensic Medicine, Tongji Medical College, Huazhong University of Science and Technology, Wuhan 430030, Hubei, China

**Keywords:** chemometrics, Fourier transform infrared spectroscopy, hierarchical cluster analysis, partial least square, Traumatic brain injury, traumatic axonal injury

## Abstract

Traumatic axonal injury (TAI) is a progressive and secondary injury following traumatic brain injury (TBI). Despite extensive investigations in the field of forensic science and neurology, no effective methods are available to estimate TAI interval between injury and death. In the present study, Fourier transform IR (FTIR) spectroscopy with IR microscopy was applied to collect IR spectra in the corpus callosum (CC) of rats subjected to TAI at 12, 24, and 72 h post-injury compared with control animals. The classification amongst different groups was visualized based on the acquired dataset using hierarchical cluster analysis (HCA) and partial least square (PLS). Furthermore, the established PLS models were used to predict injury interval of TAI in the unknown sample dataset. The results showed that samples at different time points post-injury were distinguishable from each other, and biochemical changes in protein, lipid, and carbohydrate contributed to the differences. Then, the established PLS models provided a satisfactory prediction of injury periods between different sample groups in the external validation. The present study demonstrated the great potential of FTIR-based PLS algorithm as an objective tool for estimating injury intervals of TAI in the field of forensic science and neurology.

## Introduction

Traumatic axonal injury (TAI) is one of the most common consequences of traumatic brain injury (TBI), accounting for 50% of TBI patients without overt mass lesions and 35% of all the deaths [1]. This type of TBI is frequently found in traffic accident, explosion, and fall cases. The major mechanism of TAI is shear and stretch injury to axons during acceleration/deceleration of the head. Some white matter bundles are more vulnerable due to their orientation and location, particularly including the corpus callosum (CC), internal capsule, optic tracts, cerebral and cerebellar peduncles, and long tracts in the brainstem [2]. Since conventional neuroimaging techniques such as CT and X-ray show no significant focal lesions [3], a definitive diagnosis of TAI is primarily dependent upon histopathology in post-mortem brain tissues. Currently, immunohistochemical detection of β-amyloid precursor protein (β-APP) has become the gold standard in evaluating TAI in both routine neuropathological and forensic settings as well as animal investigations [4–7]. In axons suffering from very rapid mechanical loadings, β-APP accumulates in the damaged sites of axons due to an impairment in axoplasmic transport.

Secondary axonal injury is predominant in TAI, which histologically evolves from early axonal swelling/varicosities, secondary disconnection, to final Wallerian degeneration [8]. From the forensic perspective, the temporal changes in TAI seemingly can been seen as a vital reaction [9], and are of specific significance for estimating survival periods following head trauma, which may help to determine the time of the incident. Although it is useful to evaluate the temporal profile of TAI based on semi-quantitation of β-APP-stained axons [10,11], this method is limited in providing an objective and high-reproducibility prediction in independent subjects. Therefore, estimating injury interval of TAI remains a challenge in forensic practice due to the lack of efficient methods.

Fourier transform IR (FTIR) spectroscopy is a non-destructive and time-saving technique for chemical analyses based on vibrational motions of various functional groups in samples. Emerging evidence shows its great potential as a new promising diagnostic method to detect chemical changes from multiple macromolecules including proteins, lipids, carbohydrates, and nucleic acids, in biological tissues [12,13]. Combination with IR microscopy allows for spectral collection in complex and heterogeneous tissues with minimal sample preparation, and the acquired spectral fingerprints can yield subsequent classification of spectra into different categories using objective computational algorithms [14,15]. According to its numerous advantages, this spectroscopic technique has been widely used in various murine brain models, including ischemic brain injury [16], Alzheimer’s disease [17], brain tumors [18], and multiple sclerosis [19], but only a few attempts have been made to study TAI [20–22].

Since TAI pathology varies with injury time, we hypothesized that these alterations could be identified in FTIR spectra and allow diagnostic segregation of TAI at different post-injury time points. While previous studies indicated the possibility of such a spectroscopic approach to identify TAI in the brainstem and CC of rats based on relative areas and intensities of the given absorption peaks [20–22], it remains difficult to distinguish TAI at different intervals depending on these limited parameters. Accordingly, the present study utilized multivariable analysis of more spectral variables, which may be more sensitive to minor spectral variations amongst different TAI time points.

Despite its importance, objective determination of injury intervals of TAI has received relatively little attention in the forensic field. To the best of our knowledge, this is the first study demonstrating the potential of FTIR spectroscopy combined with chemometrics in estimating TAI intervals post-injury. In the current study, spectral information was acquired in the CC of rats subjected to TAI, at 12, 24, and 72 h post-injury. The resulting spectral data were classified by hierarchical cluster analysis (HCA) and partial least square (PLS). Subsequently, differentiation of TAI intervals in independent samples was performed by the established PLS models.

## Materials and methods

### TAI animal model

The ethics committee of Xi’an Jiaotong University specifically approved the present study. Male Sprague–Dawley rats weighing 280–320 g were socially housed under a 12-h light/dark cycle with food and water *ad libitum*. The animals were divided into four different groups, including control, 12, 24, and 72 h injury groups (15 rats per group). For each group, ten animals were used for HCA and PLS calibration, and the classification of the remaining five animals was performed using the developed PLS models.

TBI was produced by a weight drop device, referred to as the Marmarou model as described previously [23,24]. Briefly, the skull was exposed between the coronal and lambdoid sutures after anesthesia with 4% isoflurane. The bone between these sutures was cleaned, dried, and a brass disc (diameter: 10 mm, thickness: 3 mm) was glued to the midline skull between bregma and lambda. Then, the animal was placed on a foam bed, with a metallic mass of 450 g freely dropped on to the center of the brass disc from a height of 2 m through a Plexiglas tube. The rat was quickly removed from the bed and ventilated with 100% O_2_ until spontaneous breathing was regained. Throughout the procedure, tail flick and foot withdrawal reflexes were monitored to ensure that an appropriate level of anesthesia was maintained, and the animal suffered minimal pain and discomfort. The incision was stitched, and rats with spontaneous respiration were immediately placed in an oxygenated and humidified chamber heated to maintain the normal body temperature. The animals were returned to the original environment after regaining complete consciousness. Control animals were anesthetized, surgically prepared, and placed under the impact device, but were not subjected to injury.

### Neurological evaluation

The severity of TBI was evaluated at different post-injury time points using the neurological severity score (NSS) after the animals regained consciousness. The NSS was used to assess both motor function and behavior. Based on previous studies [25–27], the test was modified appropriately in the present study, which consisted of ten individual clinical parameters (Table 1). The parameters were scored on a scale from 0 (no deficit) to 10 (maximal deficit). For NSS data, Prism 5.0 (GraphPad Software Inc., La Jolla, CA) was used to perform one-way ANOVA. When ANOVA yielded statistical significance, it was followed by a Dunnett’s post hoc analysis to assess the differences amongst the control and injury groups at different time points. *P*<0.05 was considered statistically significant. Data are mean ± S.D..

**Table 1:** NSS presented in the study

Task	Description	Points (success/failure)
Exit circle	Ability and initiative to exit a circle of 50 cm diameter within 3 min	0/1
Monoparesis/hemiparesis	Paresis of upper and/or lower limb of the contralateral side	1/0
Straight walk alertness	Initiative and motor ability to walk straight	0/1
Startle reflex	Innate reflex; the mouse will bounce in response to a loud hand clap	0/1
Seeking behavior	Physiological behavior as a sign of ‘interest’ in the environment	0/1
Beam balancing	Ability to balance on a beam of 1.5-cm width for at least 20 s	0/1
Round stick balancing	Ability to balance on a round stick of 1-cm diameter for at least 20 s	0/1
Beam walk: 8.5 cm	Ability to cross a 30-cm long beam of 6-cm width	0/1
Beam walk: 5 cm	Same task, increased difficulty on a 4-cm wide beam	0/1
Beam walk: 2.5 cm	Same task, increased difficulty on a 2-cm wide beam	0/1
Maximum score		10

### Immunohistochemistry

Brain tissues were fixed in 4% paraformaldehyde for 24 h. The cerebral block was trimmed to include the CC under the impact center, immersed in 25% sucrose, and embedded in an optimal cutting tool. Serial 10-μm thick coronal sections were obtained with a cryotome and mounted on glass slides. The acquired brain sections were incubated in 3% hydrogen peroxide to quench endogenous peroxidase for 15 min. After washing with 0.01 M PBS, the sections were incubated with 5% BSA for 60 min to reduce non-specific reactions, and treated with rabbit β-APP monoclonal antibody at 1:4000 dilution (Abcam, ab32136, Cambridge, U.K.) at 4°C overnight. Biotinylated goat anti-rabbit secondary antibody and streptavidin–biotin complex reagent (Boster, Wuhan, China) were applied subsequently at 37°C for 45 min, respectively. Positive reactions were visualized with diaminobenzidine. The sections were dehydrated in graded alcohol, cleared in xylene, and mounted.

### FTIR data collection and pre-processing

Brain sections from the same animal were mounted on IR transparent slides (barium fluoride, BaF_2_). To eliminate the interference of moisture, brain sections were dried for 30 min before FTIR data collection. Spectral measurements were performed on an FTIR spectrometer (Thermo Scientific Nicolet TM 5700-II, MIT, U.S.A.) coupled to IR microscopy (Nicolet Continue μmXL, MIT, U.S.A.) with a liquid nitrogen cooled mercury cadmium telluride (MCT) detector. Each spectrum was obtained at a resolution of 16 cm^−1^ and eight scans with a frequency of 3300–900 cm^−1^. The collective aperture of the IR microscopy was set to 60 × 60 μm. The regions of interest for spectral collection were determined by comparison with immunohistochemical results in corresponding sections.

FTIR spectral collection was conducted on an OMNIC Picta 8.0 (Thermo Fisher, U.S.A.), with data pre-processing performed with MATLAB 2014a (MathWorks, U.S.A.) equipped with PLS Toolbox 8.1.1 (Eigenvector Research, Inc., USA). Raw spectra were pre-processed by smoothing, baseline correction, and normalization by multiplicative signal correction (EMSC). To evaluate within-group variations, mean spectrum with S.D. was derived for a given group. Supplementary Figure S1 shows mean spectra with corresponding S.D. in all the groups; minor within-group differences in each group were found, mainly occurring in the fingerprint regions (1400–900 cm^−1^).

### Multivariable statistical analysis

In the present study, multivariable statistical analysis included HCA and PLS, both of which were conducted with MATLAB 2014a (MathWorks, U.S.A.) equipped with PLS Toolbox 8.1.1 (Eigenvector Research, Inc., U.S.A.). The predictor variable X was composed of the spectral intensity matrix at 1800–900 cm^−1^, while the response variable Y corresponded to dummy variables such as 1, 2, and 3, which represented different groups.

HCA used the agglomerative method to identify clusters in the calibration dataset. In this process, samples with spectral variables in close proximity were linked together, until all clusters were joined into one large group. HCA was performed using Euclidean Distance as the proximity measure and Complete Linkage as amalgamation strategy. HCA results were visualized graphically by a 2D tree diagram known as dendrogram.

PLS algorithm can extract principle components (referred to as latent factors) simultaneously from the predictor variable X and response variable Y for model construction. The spectral dataset in the calibration group was used to calibrate the PLS model through leave-one-out cross-validation (LOOCV). Data were interpreted by the score plot and variable importance in the projection (VIP). In the PLS score plot, all spectra were projected on a 2D graph in the form of score points, thus allowing visualization of the classification amongst groups. VIP values were the weighed sums of squares of PLS weights [28], and can be used to evaluate the contribution of spectral variables into the classification. The variables with VIP values above 1.0 were considered significant. Then, the spectral dataset in the validation group was classified by the established PLS models in order to assess the predictive performance.

## Results and discussion

**Figure 1:**
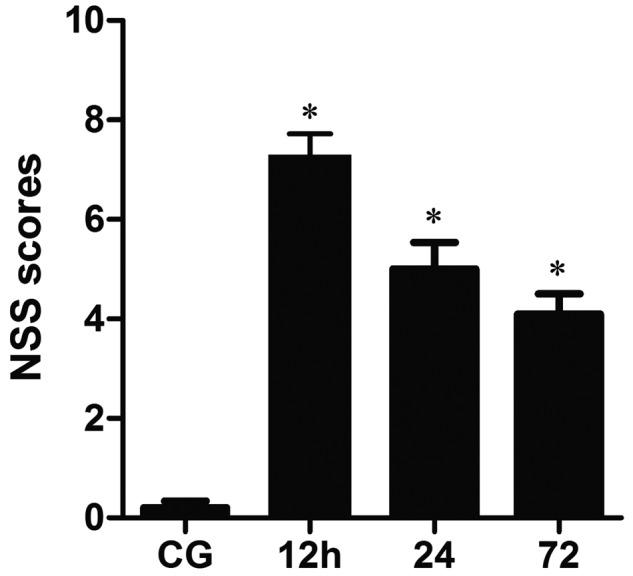
The changes in NSS at 12, 24, 72 h post-injury and in the CG Significant increases occur in the average NSS following TBI in contrast with CG, but a progressive decrease from 12 to 72 h post-injury (*P<0.05).

**Figure 2:**
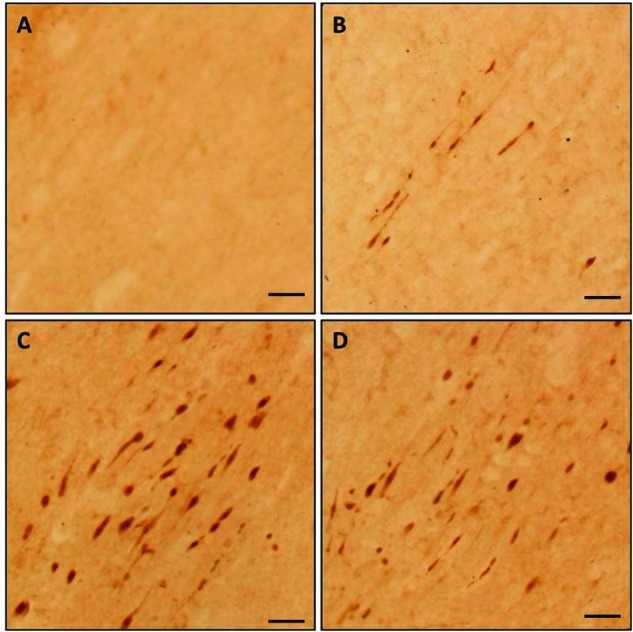
Axonal injury in the CC detected by β-APP staining following impact acceleration of TBI. (**A**) No β-APP staining is found in the CG. Few immunoreactive profiles are observed at 12 h post-injury, (**B**) while there are numerous β-APP-stained axons found at 24 (**C**) and 72 h post-injury (**D**), but is more evident at 24 h (scale bar =50 μm).

Since, the CC is one of the predilection sites for TAI in human and animal brains, investigation is mainly focussed on this region for FTIR analysis. In the present study, the Marmarou model was used to mimic TAI in humans, as it is well established that this model can produce widespread damage in neurones, axons, and microvasculature, especially in the CC [23,24]. Before FTIR analysis, the clinical status of the injured rats was evaluated by the modified NSS test. Figure 1 presents NSS data in control and injury groups at different post-injury intervals. In contrast with control group (CG), average NSS values in injury groups were significantly increased but with a progressive decrease across all time points. This suggests that the loading impact leads to severe TBI, which is gradually alleviated over time; this may be associated with axonal recovery post-injury. β-APP immunochemistry was used to confirm the presence of axonal injury in the CC. As shown in Figure 2, no positive staining was detected in the CG, while β-APP stained axons appearing as dot-like or swelling profiles were found at all the time points, with the 24-h group showing more evident changes. The temporal histological findings are consistent with previously published studies although the animal model and anatomic sites are different [1,29].

Matching with β-APP immunochemistry, the positive-stained axons were mainly concentrated in the midline of coronal sections of the CC under the loading impact, especially at 24 h (Figure 2C) and 72 h (Figure 2D) post-injury while there were negative axons in the normal group (Figure 2A); thus, this area was determined as the region of interest in the present study. Nevertheless, only a few injured axons were identified within this area at 12 h post-injury as shown in Figure 2B. It was proposed that other seemingly normal axons may also suffer secondary pathophysiological changes and contribute to axonal dysfunction [30–32]. Accordingly, spectral information at 12 h post-injury was still collected in the same region of interest used for the 24- and 72-h groups, even though most negative-stained axons were included.

**Figure 3:**
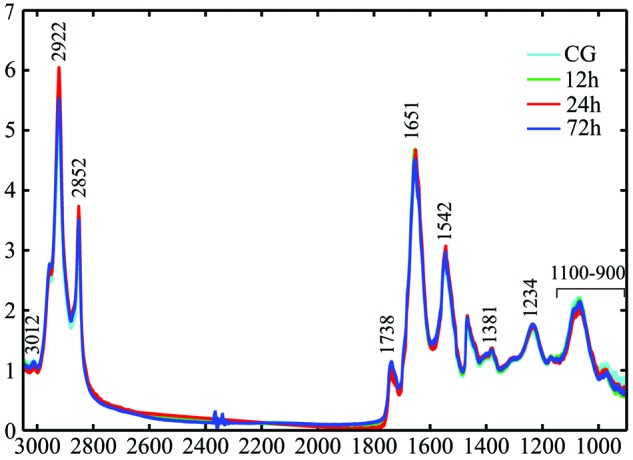
Superimposition of all normalized spectra collected in the CC of rats at different post-injury groups. The absorption bands representing various chemical functional groups are marked.

All normalized spectra of TAI rats acquired at different post-injury time points are displayed in Figure 3, where several absorption peaks could be identified, corresponding to different chemical functional groups. Within the frequency range of 2800–3100 cm^−1^, the main contributors are lipids due to C–H vibrations from CH_2_ (asymmetric and symmetric vibrations at 2922 and 2852 cm^−1^, respectively), CH_3_ (asymmetric vibration at 2958 cm^−1^), and C=C–CH_2_ (3012 cm^−1^) [33]. The band at 1738 cm^−1^ is due to C=O vibrations from lipid esters [34,35]. Amide I (1652 cm^−1^) and II (1542 cm^−1^) bands mainly originate from C=O and N–H vibrations in proteins [36–38]. The band centered at 1380 cm^−1^ is related to the symmetric bending mode of CH_3_ in proteins. P–O asymmetric stretching in phospholipids contributes to the band at 1235 cm^−1^ [39]. In the range of 1100–900 cm^−1^, carbohydrates are the main contributors [40].

At first glance, spectra from different groups were very similar but major differences were clearly found between the 24 h and other groups. In terms of lipids, the 24-h group had higher intensities at 2922 and 2852 cm^−1^, but a lower one at 2958 cm^−1^. There were also significant changes in unsaturated lipids at this time point as revealed by lower intensities at 3012 and 1738 cm^−1^. Consistent with the histological examination, the observed spectral pattern implies that axonal injury in the CC is more serious at 24 h following closed head injury, accompanied by significant biochemical changes associated with lipids and proteins. Although these findings demonstrate that multiple chemical components in the CC of rats can be discriminated by FTIR spectroscopy, it remains difficult to distinguish different TAI time points depending on quantitation of the given absorption peaks. Therefore, multivariable statistical analysis, including HCA and PLS, is required to identify subtle spectral variations and to visualize the classification amongst the groups in the present study.

**Figure 4:**
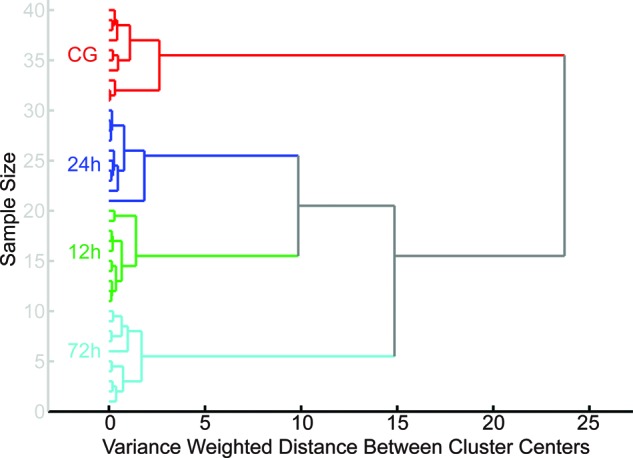
The classification result of HCA dendrogram amongst CG, 12, 24, and 72 h post-injury groups.

**Figure 5:**
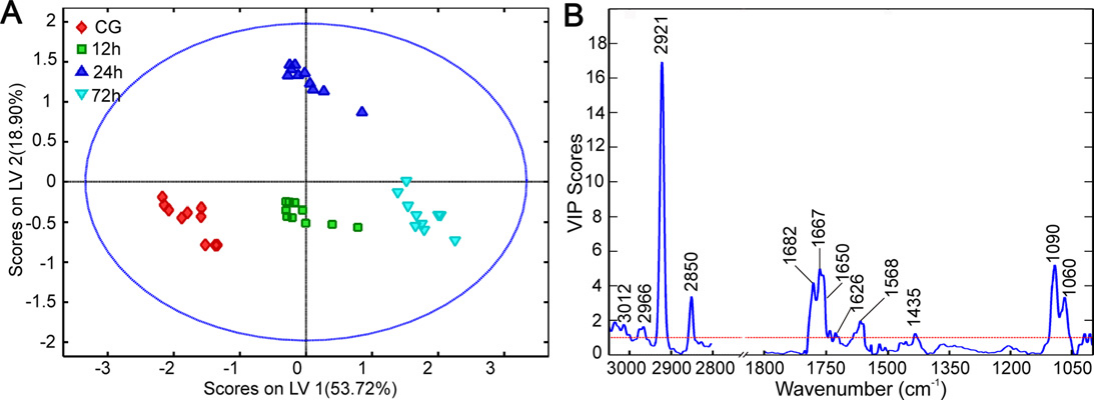
The classification result of PLS algorithm amongst CG, 12, 24, and 72 h post-injury groups. (**A**) PLS scores plot, (**B**) the plot of VIP scores as a function of wave number.

HCA was first performed on all spectral variables in the calibration group within the ranges of 3100–2800 cm^−1^ and 1800–900 cm^−1^. The HCA dendrogram (Figure 4) shows that samples from the same category were successfully grouped and closely linked to each other, and four main clusters were obtained based on different sample groups. These results demonstrate that classification amongst TAI intervals could be achieved based on the FTIR spectral signature collected in the CC. Accordingly, this helped us to establish a mathematical algorithm according to the FTIR dataset for predicting TAI survival intervals. In the present study, the PLS model was established based on the calibration dataset and used to predict validation samples. Numerous studies have confirmed the efficiency of this supervised model in classifying or predicting different categories of biological tissues [41,42,14]. In Figure 5A, the PLS score plot illustrates a straightforward separation amongst the four groups, where the control, 12, and 24h groups were distributed along LV1 accounting for 53.72% of total variance, while 24 h and the remaining groups were segregated along LV2 (18.90% of total variation). In agreement with HCA, this finding indicates that most spectral variations are responsible for the segregation amongst the groups. To assess whether these variables contributing to the distinction primarily originate from chemical components in the CC rather than reflecting an artifact effect, VIP values for all variables were calculated as a function of wave number. As shown in Figure 5B, the most influential absorption peaks mainly arose from lipids (3100–2800 cm^−1^), proteins (1700–1500 cm^−1^), and carbohydrates (1100–900 cm^−1^). Notably, VIP calculation allows the resolution of overlapping bands included in the Amide I band, where some absorption peaks associated with protein conformation are considered important contributors for the spectral differences amongst various groups, including β-turn (1682 cm^−1^), β-turn (1667 cm^−1^), α-helix (1652 cm^−1^), and β-sheet (1626 cm^−1^) [39,43].

Growing evidence shows that multiple pathways and pathophysiological processes are involved in axonal degeneration of TAI and differ with time post-injury, resulting in a spectrum of content and structural alterations in the white matter. Our FTIR investigation showed that biochemical changes in the CC involving proteins, lipids, and carbohydrates contributed to axonal pathology with the development of injury interval. In terms of lipids, notable differences in CH_3_, CH_2_, C=C–C, and C=O contents are the major discriminators for the classification of TAI groups at different time points. These spectral features provide information on the length and unsaturation of acyl chains. This variation may be interpreted by lipid peroxidation following TBI, which is associated with both mitochondrial dysfunction and cytoskeletal degradation *in vivo* [44–48]. Since lipid peroxidation primarily occurs at double-bond sites of polyunsaturated acyl chains, there is an increase in CH_3_ content with notable reduction in CH_2_ and double bonds (C=C–C and C=O); however, loss of unsaturation is compensated by the accumulation of double bonds in lipid peroxidation end products [49]. In addition, our study demonstrated that significant alterations of protein conformation are responsible for such distinction, including α-helix, β-sheet, and β-turn structures. Recent findings in our laboratory proposed that α-helix structures are predominant in the normal white matter while β-structures are prominent following TAI; this may be due to the denaturation of axonal cytoskeleton and deposit of axoplasmic proteins rich in β-structures [7]. In the latter study, it was notable that these differences in protein conformation are only found at 24 h post-injury using curve fitting methods. Thus, the present study confirmed that use of multivariable analysis is more sensitive for subtle spectral variations amongst different groups.

Other wave numbers responsible for the separation were correlated with C–O vibrations from carbohydrates within the frequency range of 1100–900 cm^−1^. Since the brain is a high energy-consuming organ, it is vulnerable to glucose and oxygen deprivation. Hyper- and hypoglycolysis are important contributors to secondary axotomy following initial injury. As reported previously, an initial brief response of hyperglycolysis occurs from hours to 5 days post-injury, which may be caused by the disruption of the blood–brain barrier (BBB) and increased glucose uptake via a mechanism that bypasses endothelial membrane glucose transporters [50]. It is possible that the abnormalities in glucose metabolism are reflected by the observed spectral variances within the frequency range of 1100–900 cm^−1^. Meanwhile, the results regarding carbohydrates imply that such alterations are time-dependent in the CC of TAI, and would be used as potential parameters for the estimation of TAI intervals.

**Figure 6:**
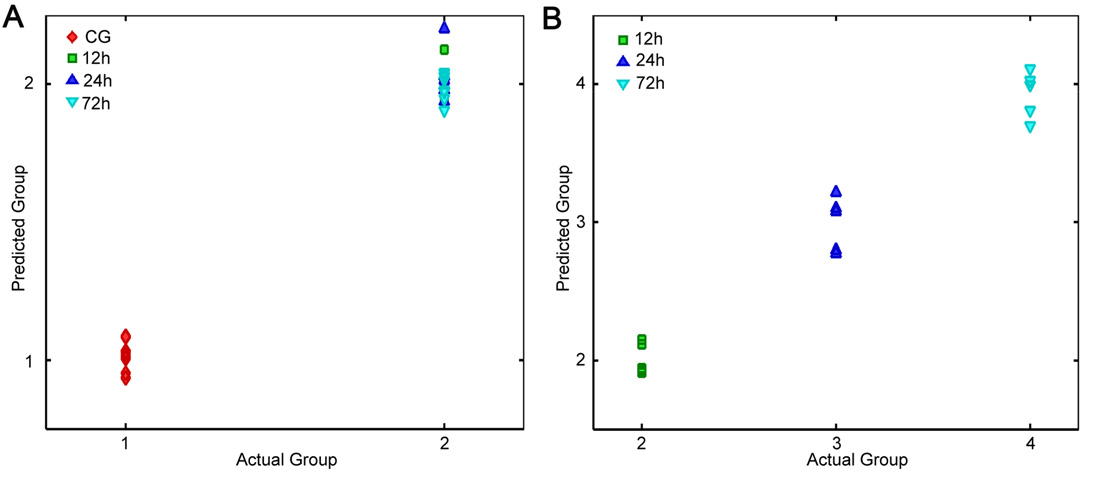
The prediction result in independent samples using the developed PLS models. The PLS model 1 is used to classify between CG and injured groups (**A**) while the model 2 is applied to distinguish amongst different TAI interval groups (**B**).

While a satisfactory classification was observed in PLS calibration, the major challenge of this model is the accurate identification of unknown samples. Therefore, the next step of the present study was to validate the performance of the developed PLS model using the spectral dataset in validation groups, which were not included for modeling. To achieve a more precise estimation, two PLS models were established, both of which were used to distinguish injury compared with CGs (model 1), and different TAI groups (model 2), respectively. Figure 6A shows that model 1 achieved a complete separation between injury and CGs, in which sample score points are close to their dummy variables (1 for CG and 2 for injury group). Subsequently, the samples in the injury group were classified by model 2, and a good prediction was obtained as shown in Figure 6B (2 for 12 h, 3 for 24 h, and 4 for 72 h).

As described above, secondary axonal injury predominates in TAI, which usually can be identified after a given survival interval following TBI. For example, secondary axotomy requires a period of >12 h in humans, but only 2–4 h in rats and cats [9]. Therefore, the evolution of axonal injury could help to estimate the survival interval in a TBI corpse. It has been found that the number of injured axons increases since initial injury, and culminates at 24 h post-injury. Despite being widely used, the major challenge of histological methods is to estimate TAI intervals post-injury as they only achieve a semi-quantitation of axonal injury, which is too subjective with low reproducibility, thus limiting their application for TAI interval prediction. Recently, there has been substantial progress using novel neuroimaging techniques that enhance the appreciation of the overall magnitude and distribution of axonal injury in TAI. Some parameters such as fraction anisotropy (FA) and axial diffusion (AF) were found to be highly correlated with TAI biomarkers [11]. Nevertheless, their feasibility in TAI interval estimation remains largely speculative. In contrast, FTIR spectroscopy with IR microscopy is known for its simplicity and sensitivity in detecting molecular and chemical changes in biological tissues. With FTIR spectroscopy, we demonstrated that different TAI intervals could be successfully classified depending on global biochemical changes of the white matter rather than the number of injured axons. Since there is no interference between FTIR analysis and histological examination, the current study provides a potential alternative method to forensic pathologists for diagnosing TAI and estimating survival intervals.

## Conclusion

In the present study, we have demonstrated for the first time that FTIR spectroscopy is a valuable approach capable of detecting temporal biochemical changes in the CC of rats subjected to TAI. Considering limitations of spatial resolution, the global chemical information was obtained within the concentration of β-APP-positive axons rather than in single injured axons such as retraction balls or swelling axons. Nevertheless, different TAI intervals were successfully classified based on the acquired spectral fingerprints using multivariable analysis, including HCA and PLS algorithms. The chemical components related to proteins, lipids, and carbohydrates greatly contributed to this distinction. Moreover, a satisfactory prediction of TAI interval was achieved in independent samples using the developed PLS models. Our study paves the way for FTIR spectroscopy with multivariable analysis to estimate the survival period of TAI. Further work should be designed to elucidate chemical changes in other predilection sites of TAI (such as the pyramidal tract and internal capsule) from human and animal samples with more time points.

## Supporting information

**Fig. S1:** The processed mean absorbance spectra from different groups with their own SDs (A: CG, B: 12h, C:24h, D: 72h). The green line is the mean spectrum for each group while the double blue lines represent its SD.

## Abbreviations

CC: corpus callosum
CG: control group
CT: computed tomography
FTIR: Fourier transform IR
HCA: hierarchical cluster analysis
LV: latent variable
NSS: neurological severity score
PLS: partial least square
TAI: traumatic axonal injury
TBI: traumatic brain injury
VIP: variable importance in the projection
β-APP: β-amyloid precursor protein

## References

[1] Bramlett H.M., Kraydieh S., Green E.J. and Dietrich W.D. (1997) Temporal and regional patterns of axonal damage following traumatic brain injury: a beta-amyloid precursor protein immunocytochemical study in rats. J. Neuropath. Exp. Neurol. 56, 1132–1141 9329457 10.1097/00005072-199710000-00007

[2] Cloots R.J., van Dommelen J.A., Kleiven S. and Geers M.G. (2013) Multi-scale mechanics of traumatic brain injury: predicting axonal strains from head loads. Biomech. Model Mechanobiol. 12, 137–150 22434184 10.1007/s10237-012-0387-6

[3] Smith D.H., Hicks R. and Povlishock J.T. (2013) Therapy development for diffuse axonal injury. J. Neurotrauma 30, 307–323 23252624 10.1089/neu.2012.2825 PMC3627407

[4] Gentleman S.M., Nash M.J., Sweeting C.J., Graham D.I. and Roberts G.W. (1993) Beta-amyloid precursor protein (beta APP) as a marker for axonal injury after head injury. Neurosci. Lett. 160, 139–144 8247344 10.1016/0304-3940(93)90398-5

[5] Sherriff F.E., Bridges L.R. and Sivaloganathan S. (1994) Early detection of axonal injury after human head trauma using immunocytochemistry for beta-amyloid precursor protein. Acta Neuropathol. 87, 55–62 8140894 10.1007/BF00386254

[6] Graham D.I., Smith C., Reichard R., Leclercq P.D. and Gentleman S.M. (2004) Trials and tribulations of using beta-amyloid precursor protein immunohistochemistry to evaluate traumatic brain injury in adults. Forensic Sci. Int. 146, 89–96 15542268 10.1016/S0379-0738(03)00274-3

[7] Zhang J., Liu L., Mu J., Yang T., Zheng N. and Dong H. (2015) Chemical analysis in the corpus callosum following traumatic axonal injury using Fourier transform infrared microspectroscopy: a pilot study. J. Forensic Sci. 60, 1488–1494 26272718 10.1111/1556-4029.12871

[8] Johnson V.E., Stewart W. and Smith D.H. (2013) Axonal pathology in traumatic brain injury. Exp. Neurol. 246, 35–43 22285252 10.1016/j.expneurol.2012.01.013 PMC3979341

[9] Finnie J.W. (2016) Forensic pathology of traumatic brain injury. Vet. Pathol. 53, 962–978 26578643 10.1177/0300985815612155

[10] Henninger N., Bouley J., Sikoglu E.M., An J., Moore C.M., King J.A. (2016) Attenuated traumatic axonal injury and improved functional outcome after traumatic brain injury in mice lacking Sarm1. Brain 139, 1094–1105 26912636 10.1093/brain/aww001 PMC5006226

[11] Li S., Sun Y., Shan D., Feng B., Xing J., Duan Y. (2013) Temporal profiles of axonal injury following impact acceleration traumatic brain injury in rats–a comparative study with diffusion tensor imaging and morphological analysis. Int. J. Legal Med. 127, 159–167 22573358 10.1007/s00414-012-0712-8

[12] Gajjar K., Trevisan J., Owens G., Keating P.J., Wood N.J., Stringfellow H.F. (2013) Fourier-transform infrared spectroscopy coupled with a classification machine for the analysis of blood plasma or serum: a novel diagnostic approach for ovarian cancer. Analyst 138, 3917–3926 23325355 10.1039/c3an36654e

[13] Dudala J., Bialas M.B., Szczerbowska-Boruchowska M., Bereza-Buziak M., Budzynski A., Hubalewska-Dydejczyk A. (2016) Investigation of biochemical composition of adrenal gland tumors by means of FTIR. Pol. J. Pathol. 67, 60–68 27179276 10.5114/pjp.2016.59221

[14] Zhang J., Li B., Wang Q., Li C., Zhang Y., Lin H. (2017) Characterization of postmortem biochemical changes in rabbit plasma using ATR-FTIR combined with chemometrics: a preliminary study. Spectrochim. Acta A Mol. Biomol. Spectrosc. 173, 733–739 27788472 10.1016/j.saa.2016.10.041

[15] Muehlethaler C., Massonnet G. and Esseiva P. (2014) Discrimination and classification of FTIR spectra of red, blue and green spray paints using a multivariate statistical approach. Forensic Sci. Int. 244, 170–178 25255193 10.1016/j.forsciint.2014.08.038

[16] Hackett M.J., Sylvain N.J., Hou H., Caine S., Alaverdashvili M., Pushie M.J. (2016) Concurrent glycogen and lactate imaging with FTIR spectroscopy to spatially localize metabolic parameters of the glial response following brain ischemia. Anal. Chem. 88, 10949–10956 27690391 10.1021/acs.analchem.6b02588

[17] Leskovjan A.C., Kretlow A. and Miler L.M. (2010) Fourier transform infrared imaging showing reduced unsaturated lipid content in the hippocampus of a mouse model of Alzheimer’s disease. Anal. Chem. 82, 2711–2716 20187625 10.1021/ac1002728 PMC2848295

[18] Beljebbar A., Amharref N., Lévèques A., Dukic S., Venteo L., Schneider L. (2008) Modeling and quantifying biochemical changes in C6 tumor gliomas by Fourier transform infrared imaging. Anal. Chem. 80, 8406–8415 18937421 10.1021/ac800990y

[19] Heraud P., Caine S., Campanale N., Karnezis T., McNaughton D., Wood B.R. (2010) Early detection of the chemical changes occurring during the induction and prevention of autoimmune-mediated demyelination detected by FT-IR imaging. Neuroimage 49, 1180–1189 19796690 10.1016/j.neuroimage.2009.09.053

[20] Reference deleted

[21] Zhang J., Niu F., Dong H., Liu L., Li J. and Li S. (2015) Characterization of protein alterations in damaged axons in the brainstem following traumatic brain injury using Fourier transform infrared microspectroscopy: a preliminary study. J. Forensic Sci. 60, 759–763 25773901 10.1111/1556-4029.12743

[22] Yang T., He G., Zhang X., Chang L., Zhang H., Ripple M.G. (2014) Preliminary study on diffuse axonal injury by Fourier transform infrared spectroscopy histopathology imaging. J. Forensic Sci. 59, 231–235 24147828 10.1111/1556-4029.12290

[23] Marmarou A., Foda M.A., van den Brink W., Campbell J., Kita H. and Demetriadou K. (1994) A new model of diffuse brain injury in rats. Part I: pathophysiology and biomechanics. J. Neurosurg. 80, 291–300 8283269 10.3171/jns.1994.80.2.0291

[24] Foda M.A. and Marmarou A. (1994) A new model of diffuse brain injury in rats. Part II: morphological characteization. J. Neurosurg. 80, 301–313 8283270 10.3171/jns.1994.80.2.0301

[25] Shapira Y., Shohami E., Sidi A., Soffer D., Freeman S. and Cotev S. (1988) Experimental closed head injury in rats: mechanical, pathophysiologic, and neurologic properties. Crit. Care Med. 16, 258–265 3277783 10.1097/00003246-198803000-00010

[26] Chen Y., Constantini S., Trembovler V., Weinstock M. and Shohami E. (1996) An experimental model of closed head injury in mice: pathophysiology, histopathology, and cognitive deficits. J. Neurotrauma 13, 557–568 8915907 10.1089/neu.1996.13.557

[27] Chen J., Sanberg P.R., Li Y., Wang L., Lu M., Willing A.E. (2001) Intravenous administration of human umbilical cord blood reduces behavioral deficits after stroke in rats. Stroke 32, 2682–2688 11692034 10.1161/hs1101.098367

[28] Chong I.-G. and Jun C.-H. (2005) Performance of some variable selection methods when multicollinearity is present. Chemometr. Intell. Lab. 78, 103–1012

[29] Marmarou C.R., Walker S.A., Davis C.L. and Povlishock J.T. (2005) Quantitative analysis of the relationship between intra- axonal neurofilament compaction and impaired axonal transport following diffuse traumatic brain injury. J. Neurotrauma 22, 1066–1080 16238484 10.1089/neu.2005.22.1066

[30] Baker A.J., Phan N., Moulton R.J., Fehlings M.G., Yucel Y., Zhao M. (2002) Attenuation of the electrophysiological function of the corpus callosum after fluid percussion injury in the rat. J. Neurotrauma 19, 587–599 12042094 10.1089/089771502753754064

[31] Reeves T.M., Phillips L.L., Lee N.N. and Povlishock J.T. (2007) Preferential neuroprotective effect of tacrolimus (FK506) on unmyelinated axons following traumatic brain injury. Brain Res. 1154, 225–236 17481596 10.1016/j.brainres.2007.04.002 PMC2703421

[32] Reeves T.M., Phillips L.L. and Povlishock J.T. (2005) Myelinated and unmyelinated axons of the corpus callosum differ in vulnerability and functional recovery following traumatic brain injury. Exp. Neurol. 196, 126–137 16109409 10.1016/j.expneurol.2005.07.014

[33] Movasaghi Z., Rehman S. and Rehman I.U. (2008) Fourier transform infrared (FTIR) spectroscopy of biological tissues. Appl. Spectrosc. Rev. 43, 134–179

[34] Kneipp J., Lasch P., Baldauf E., Beekes M. and Naumann D. (2000) Detection of pathological molecular alterations in scrapie-infected hamster brain by Fourier transform infrared (FT-IR) spectroscopy. Biochim. Biophys. Acta 1501, 189–199 10838192 10.1016/s0925-4439(00)00021-1

[35] Gasper R., Dewelle J., Kiss R., Mijatovic T. and Goormaghtigh E. (2009) IR spectroscopy as a new tool for evidencing antitumor drug signatures. Biochim. Biophys. Acta 1788, 1263–1270 19250921 10.1016/j.bbamem.2009.02.016

[36] Kos G., Krska R., Lohninger H. and Griffiths P.R. (2004) A comparative study of mid-infrared diffuse reflection (DR) and attenuated total reflection (ATR) spectroscopy for the detection of fungal infection on RWA2-corn. Anal. Bioanal. Chem. 378, 159–166 14551669 10.1007/s00216-003-2245-y

[37] Stone N., Kendall C., Smith J., Crow P. and Barr H. (2004) Raman spectroscopy for identification of epithelial cancers. Faraday Discuss. 126, 141–157 14992404 10.1039/b304992b

[38] Faolain E.O., Hunter M.B., Byrne J.M., Kelehan P., Lambkin H.A., Byrne H.J. (2005) Raman spectroscopic evaluation of efficacy of current paraffin wax section dewaxing agents. J. Histochem. Cytochem. 53, 121–129 15637345 10.1177/002215540505300114

[39] Caine S., Heraud P., Tobin M.J., McNaughton D. and Bernard C.C. (2012) The application of Fourier transform infrared microspectroscopy for the study of diseased central nervous system tissue. Neuroimage 59, 3624–3640 22119649 10.1016/j.neuroimage.2011.11.033

[40] Peng C., Chiappini F., Kascakova S., Danulot M., Sandt C., Samuel D. (2015) Vibrational signatures to discriminate liver steatosis grades. Analyst 140, 1107–1118 25581590 10.1039/c4an01679c

[41] Mistek E. and Lednev I.K. (2015) Identification of species’ blood by attenuated total reflection (ATR) Fourier transform infrared (FT-IR) spectroscopy. Anal. Bioanal. Chem. 407, 7435–7442 26195028 10.1007/s00216-015-8909-6

[42] Khoshmanesh A., Dixon M.W., Kenny S., Tilley L., McNaughton D. and Wood B.R. (2014) Detection and quantification of early-stage malaria parasites in laboratory infected erythrocytes by attenuated total reflectance infrared spectroscopy and multivariate analysis. Anal. Chem. 86, 4379–4386 24694036 10.1021/ac500199x PMC4014274

[43] Lu R., Li W.W., Katzir A., Raichlin Y., Yu H.Q. and Mizaikoff B. (2015) Probing the secondary structure of bovine serum albumin during heat-induced denaturation using mid-infrared fiberoptic sensors. Analyst 140, 765–770 25525641 10.1039/c4an01495b

[44] Deng Y., Thompson B.M., Gao X. and Hall E.D. (2007) Temporal relationship of peroxynitrite-induced oxidative damage, calpain-mediated cytoskeletal degradation and neurodegeneration after traumatic brain injury. Exp. Neurol. 205, 154–165 17349624 10.1016/j.expneurol.2007.01.023 PMC1950332

[45] DeKosky S.T., Abrahamson E.E., Ciallella J.R., Paljug W.R., Wisniewski S.R., Clark R.S. (2007) Association of increased cortical soluble abeta42 levels with diffuse plaques after severe brain injury in humans. Arch. Neurol. 64, 541–544 17420316 10.1001/archneur.64.4.541

[46] Fujita M., Oda Y., Wei E.P. and Povlishock J.T. (2011) The combination of either tempol or FK506 with delayed hypothermia: implications for traumatically induced microvascular and axonal protection. J. Neurotrauma 28, 1209–1218 21521034 10.1089/neu.2011.1852 PMC3136741

[47] Mustafa A.G., Singh I.N., Wang J., Carrico K.M. and Hall E.D. (2010) Mitochondrial protection after traumatic brain injury by scavenging lipid peroxyl radicals. J. Neurochem. 114, 271–280 20403083 10.1111/j.1471-4159.2010.06749.x PMC3526891

[48] Mustafa A.G., Wang J.A., Carrico K.M. and Hall E.D. (2011) Pharmacological inhibition of lipid peroxidation attenuates calpain-mediated cytoskeletal degradation after traumatic brain injury. J. Neurochem. 117, 579–588 21361959 10.1111/j.1471-4159.2011.07228.x PMC3076544

[49] Cakmak G., Miller L.M., Zorlu F. and Severcan F. (2012) Amifostine, a radioprotectant agent, protects rat brain tissue lipids against ionizing radiation induced damage: an FTIR microspectroscopic imaging study. Arch. Biochem. Biophys. 520, 67–73 22402174 10.1016/j.abb.2012.02.012

[50] Lou M., Zhang H., Wang J., Wen S.Q., Tang Z.Q., Chen Y.Z. (2007) Hyperbaric oxygen treatment attenuated the decrease in regional glucose metabolism of rats subjected to focal cerebral ischemia: a high resolution positron emission tomography study. Neuroscience 146, 555–561 17367940 10.1016/j.neuroscience.2007.01.046

