# Supplementary material for: Application of FTIR spectroscopy for traumatic axonal injury: a possible tool for estimating injury interval
Source: Biosci Rep. 2017 Jul 21;37(4):BSR20170720. doi: 10.1042/BSR20170720 (PMC5567294; doi:10.1042/BSR20170720)
Supplement: Supplementary file 1 [file bsr-37-bsr20170720-s1.pdf]

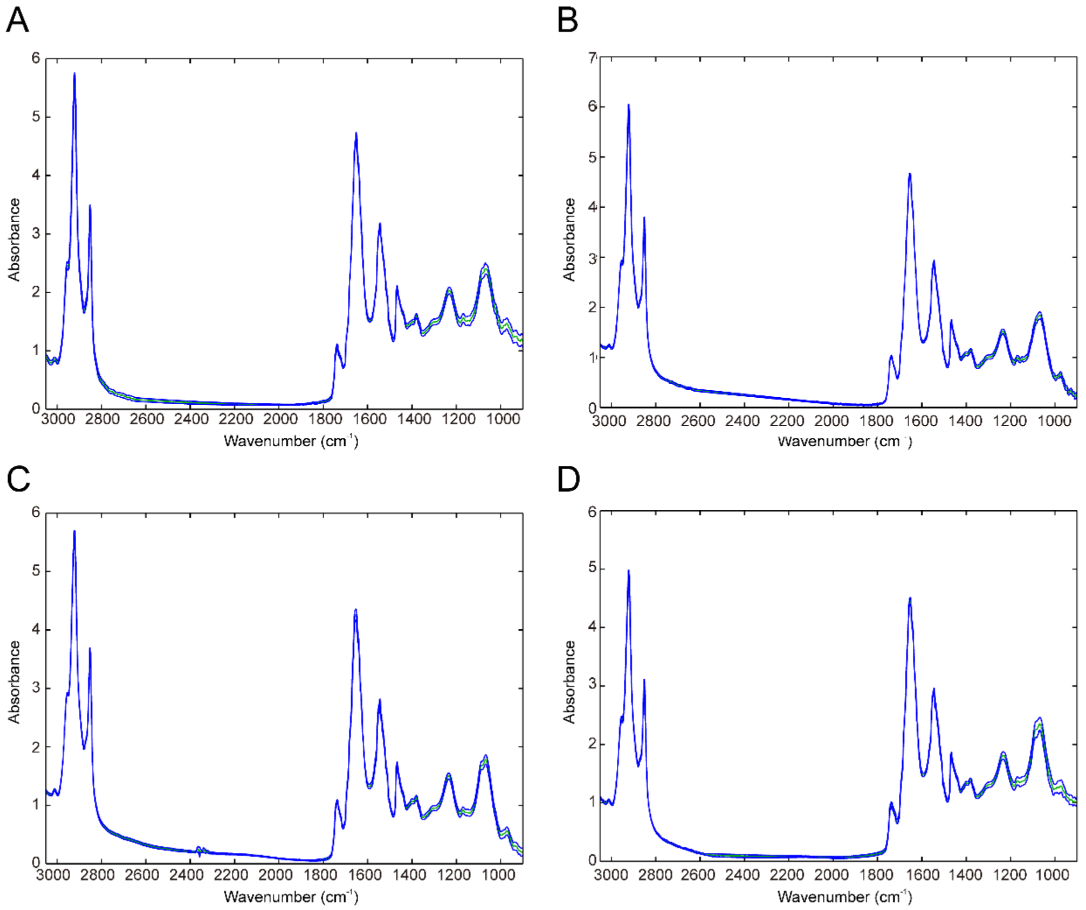

Fig. S1 The processed mean absorbance spectra from different groups with their own SDs (A: CG, B: 12h, C:24h, D: 72h). The green line is the mean spectrum for each group while the double blue lines represent its SD.
